# Mechanisms of Ectopic Gene Conversion

**DOI:** 10.3390/genes1030427

**Published:** 2010-11-29

**Authors:** P.J. Hastings

**Affiliations:** Department of Molecular and Human Genetics, Baylor College of Medicine, 1 Baylor Plaza, Houston, TX 77030, USA; E-Mail: hastings@bcm.edu; Tel.: +1-713-798-5787

**Keywords:** double-strand break, recombination, crossover, gene conversion, ectopic gene conversion, SDSA, BIR, synaptonemal complex, cohesin, mismatch repair

## Abstract

Gene conversion (conversion), the unidirectional transfer of DNA sequence information, occurs as a byproduct of recombinational repair of broken or damaged DNA molecules. Whereas excision repair processes replace damaged DNA by copying the complementary sequence from the undamaged strand of duplex DNA, recombinational mechanisms copy similar sequence, usually in another molecule, to replace the damaged sequence. In mitotic cells the other molecule is usually a sister chromatid, and the repair does not lead to genetic change. Less often a homologous chromosome or homologous sequence in an ectopic position is used. Conversion results from repair in two ways. First, if there was a double-strand gap at the site of a break, homologous sequence will be used as the template for synthesis to fill the gap, thus transferring sequence information in both strands. Second, recombinational repair uses complementary base pairing, and the heteroduplex molecule so formed is a source of conversion, both as heteroduplex and when donor (undamaged template) information is retained after correction of mismatched bases in heteroduplex. There are mechanisms that favour the use of sister molecules that must fail before ectopic homology can be used. Meiotic recombination events lead to the formation of crossovers required in meiosis for orderly segregation of pairs of homologous chromosomes. These events result from recombinational repair of programmed double-strand breaks, but in contrast with mitotic recombination, meiotic recombinational events occur predominantly between homologous chromosomes, so that transfer of sequence differences by conversion is very frequent. Transient recombination events that do not form crossovers form both between homologous chromosomes and between regions of ectopic homology, and leave their mark in the occurrence of frequent non-crossover conversion, including ectopic conversion.

## 1. Introduction

Gene conversion, also called conversion, is the non-reciprocal transfer of genetic information between regions of DNA. Sequence information is transferred from one molecule, the donor, to another molecule with which it shares sequence similarity, the recipient. The donor molecule is usually unchanged in this reaction. Conversion is of considerable importance because it constitutes the main method of recombining allelic differences within genes, and serves to keep diverging sequences similar. 

In discussion of sequence similarity, different levels of similarity are distinguished. Sequence identity refers to a length of sequence in which there is no difference between two copies. Sequence homology is arbitrarily defined as greater than 97 or 98% identity. In other words, there might be up to two or three allelic differences per 100 base-pairs. Sequence with less than 97% identity is homeologous sequence, and unrelated sequence is heterologous. 

Conversion is a byproduct of recombinational repair mechanisms. When one strand of a DNA molecule is damaged, repair mechanisms can replace damaged sequence by synthesizing the complement of the intact strand. However, when both strands are damaged, for example by a double-strand break (DSB) or a non-replicating lesion opposite to a single-strand gap, sequence information to repair the damage might be derived from homologous sequence in another DNA molecule or region. 

The most obvious intact homologous sequence would be the sister chromatid in a cell in S or G2 phases of the cell cycle. Recombination with a sister molecule leaves no genetic trace because there are no sequence differences. Homology is also available in the form of homologous chromosome pairs in diploid cells. The use of a homologue for repair can lead to homozygosis, or loss of heterozygosity, by converting one allele to the form of that in the homologous chromosome. Homology is also found ectopically in repeated sequences, either within a chromosome or in other heterologous chromosomes. 

Conversion is sometimes accompanied by a crossover, that is, a reciprocal exchange between two molecules. Such exchanges will recombine all sequence centromere-distal to the crossover with all sequence proximal to it (unless another crossover intervenes). Crossing-over might be disadvantageous when it occurs in somatic cells, because it can lead to loss of heterozygosity when it involves homologous chromosomes, and to chromosomal structural changes such as inversions, duplications, deletions, translocations and the formation of dicentric and acentric chromosomes when ectopic homology is involved. As described below, the major recombination mechanisms that occur in somatic cells do not often lead to crossing-over. 

Programmed recombination, including crossing-over and conversion, plays major roles in the generation of antibody variability, and in meiosis where, in most eukaryotes, crossovers form the chiasmata that hold homologues together to ensure orderly segregation in the first meiotic division. Conversion accompanies these crossovers and leads to frequent recombination between allelic differences within genes. In addition, numerous non-crossover events show conversion between homologous chromosomes and between regions of ectopic homology. As will become apparent below, conversion does not transfer sequence information at a point. Rather, a length of sequence information is transferred. This length can be quite short, especially in meiosis, often less than the length of a gene, but can extend for tens of kilobase pairs in mitotic yeast cells. 

The mechanisms described below reflect our current understanding of the processes based on the evidence available. We do not expect that the mechanisms are fully understood, but these working models provide adequate structures on which to base our thinking. 

## 2. Conversion as a Consequence of DNA Repair

### 2.1. Double-Strand Break-Repair

**Figure 1:**
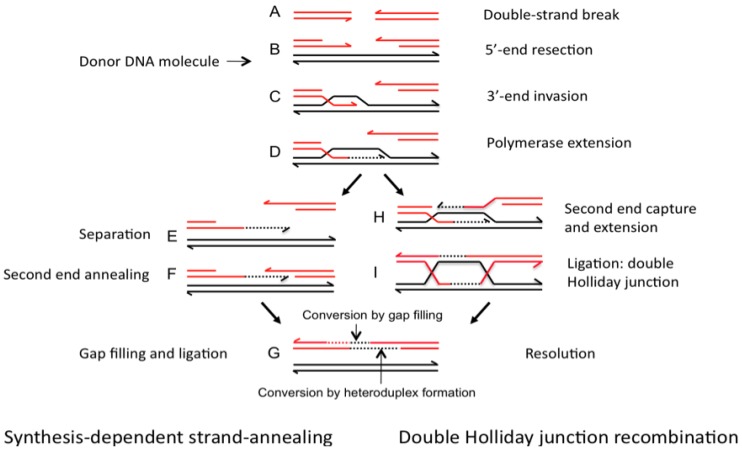
Mechanisms of gene conversion by double-strand break repair. The ends of a broken DNA molecule (**A**) are processed by exonucleolytic resection of the 5' end to give a 3' overhanging tail (**B**). This tail is coated by the Rad51 recombinase and then invades a homologous donor sequence forming a D-loop (**C**). The 3'-end in the D-loop primes DNA synthesis that copies the homologous sequence (**D**). At this point there are two different possible routes: In a pathway called synthesis-dependent strand-annealing (SDSA), the extended 3'-end is dissociated from the D-loop (**E**). It might then anneal to the 3' tail from the other side of the break, because the extended sequence is now complementary to that 3'-end (**F**). After gap-filling and ligation we are left with an unchanged donor molecule and a repaired molecule that carries sequence copied from the donor (**G**). Where the site of the original break was bridged, donor sequence is in both strands. Where sequence copied from the donor has annealed to the other 3'-end, there is heteroduplex, having one strand from the donor sequence and one from the damaged molecule. Resolution of heteroduplex is described in Figure 2. SDSA is the major mode of recombinational repair of double-strand breaks in somatic cells. It does not lead to crossing over. In an alternative process called the double Holliday junction pathway, the 3'-end from the other side of the break anneals to the displaced strand in the D-loop (**H**), generating a double Holliday junction (**I**). As shown, the double Holliday junction can be resolved by endonuclease or by helicase and topoisomerase to give the same non-crossover configuration (G). Alternatively, the double Holliday junction can be resolved endonucleolytically to form a cross-over (not shown). Each line represents a nucleotide chain or strand of DNA. The damaged molecule is shown as red and the donor (undamaged) molecule, as well as sequences copied from it, are shown in black. New DNA synthesis is shown as dotted lines. Polarity is indicated by half arrows on 3' single-strand ends.

Double-strand break repair has been reviewed extensively [1,2,3,4]. This recombination repair mechanism has been well studied in yeast and human, because of the ease with which breaks can be induced *in vivo* by site-specific endonucleases. The mechanisms that lead to conversion are illustrated in Figure 1. In vegetative cells the preferred mechanism, illustrated on the left of Figure 1, is called synthesis-dependent strand-annealing (SDSA). The broken ends are prepared by nucleolytic resection of the 5'-ends, so that long 3'-tails are produced. The 3'-ending strands become coated with Rad51 protein that catalyzes a search for homology, invasion of homologous duplex DNA by the Rad51-coated single strand, and displacement of the like DNA strand. This structure is called a D-loop. The 3'end in the D-loop primes DNA synthesis, using the complementary strand of the invaded molecule (the donor) as a template. This has the effect of extending the 3'-end past the position of the original DSB, thus allowing the break to be bridged. This synthesis is of low processivity, and stops after a short length has been produced. The extended 3'-end then separates from the D-loop and anneals with the processed 3'-end from the other side of the break, because it now carries sequence complementary to that end (Figure 1F). Gaps in both strands are filled using the other strand as a template, and ligation restores an intact repaired molecule. The donor molecule is unchanged by these events. 

**Figure 2:**
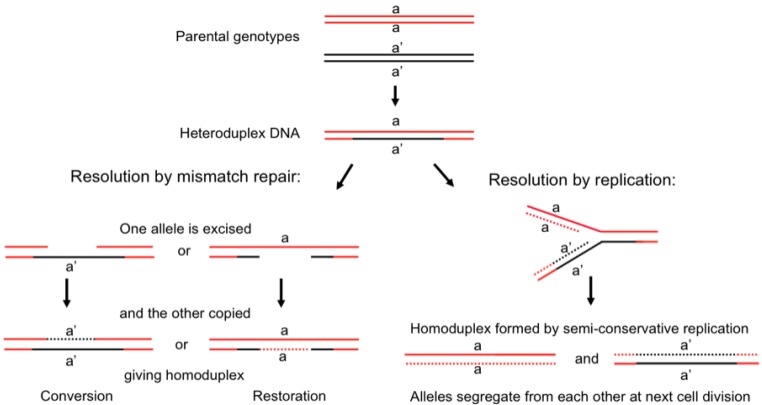
Mechanisms of conversion from heteroduplex DNA. Recombination forms hybrid DNA molecules with two complementary strands from different sources, shown here as a length of black (donor) sequence in the red recipient molecule. If there is an allelic difference (a or a’), the hybrid molecule is called heteroduplex. Heteroduplex is subject to mismatch repair (left) by which a length of one strand including the mismatch is excised and replaced by copying the other strand. If the recipient (red) strand is excised, the recipient molecule will be converted to the genotype of the donor (black). If the donor sequence is excised, the genotype of the recipient molecule will be restored. Heteroduplex DNA can also be resolved by replication (right) resulting in conversion in one of two daughter cells. Each line represents a single strand of DNA. New synthesis is shown as a dotted line.

SDSA in mitotic cells shows a very strong preference for the sister chromatid as a template for repair, so that it does not usually lead to genetic change. Rarely a homologous chromosome or length of homology in a non-allelic position is used. If there are allelic differences between the sequences, conversion can result from two mechanisms as shown in Figure 1. First, if there was a double-strand gap where the break occurred, this will be filled by sequence with the genotype of the donor molecule. Second, when sequence copied from the donor anneals with the broken molecule, an allelic difference will result in a base-pair mismatch. Such a molecule is called heteroduplex DNA. Heteroduplex is subject to an excision repair process called mismatch repair (MMR), as illustrated in Figure 2, in which one strand is excised over a length and replaced by copying the other strand. Removal of the mismatched segment of the donor strand restores the genotype of the recipient, whereas excision of the recipient strand converts the recipient to the genotype of the donor. Conversion also occurs in half the daughter cells if the mismatch in the heteroduplex DNA is not corrected but is resolved by subsequent replication (Figure 2). 

The other mechanism of homologous recombination using a double Holliday junction (Figure 1, right) has been understood in outline for a long time, but does not have a generally accepted name and is referred to (mistakenly) as gene conversion, or as canonical double-strand break-repair or double-Holliday junction (dHJ) recombination. A double Holliday junction is formed by incorporation of the 3'-end from the other side of the break into the D-loop. The double Holliday junction is often resolved by endonuclease into a crossover. As shown, the structure has been resolved by endonuclease or by helicase and topoisomerase [5] to generate the same non-crossover structure as is obtained by SDSA. A DSB is ten-times less likely to be repaired by dHJ recombination in mitotic yeast cells than in meiotic cells, making SDSA the predominant mechanism in mitotic cells [6].

Figure 1 and Figure 2 show that, whether stemming from gap-filling or from mismatch correction of heteroduplex, conversion does not occur at a point, but changes a length of DNA sequence. An important consequence of this is that two or more allelic differences can be co-converted in the same direction at the same time. Intragenic recombination is thus less common than the rate at which that region is converted. Intragenic recombination requires that a conversion tract should end between the alleles. Lengths of co-conversion can cover tens of kilobase pairs of sequence in mitotic cells. 

### 2.2. Other DNA Repair Mechanisms

**Figure 3:**
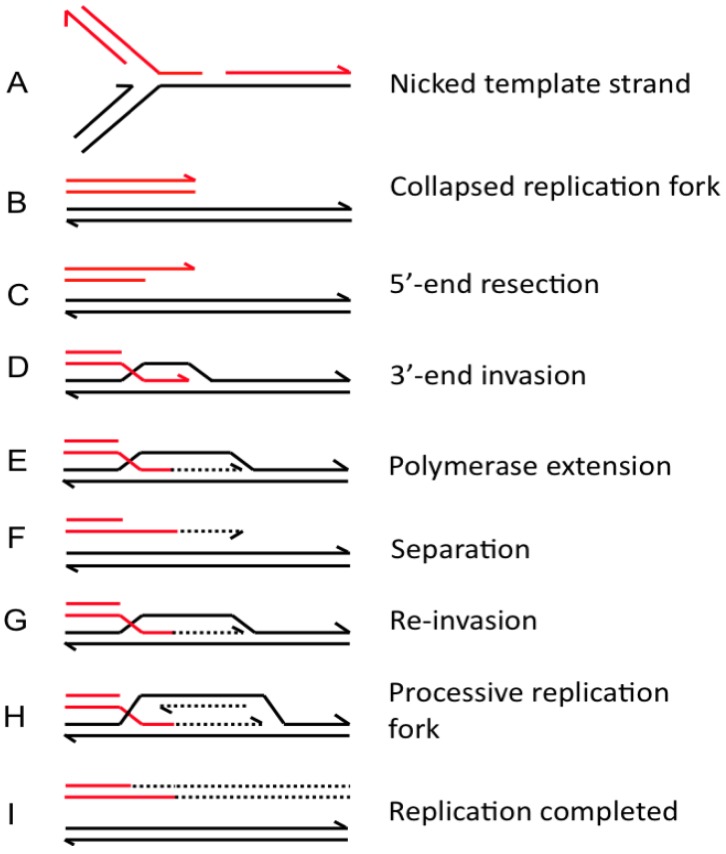
Repair of a collapsed replication fork by break-induced replication (BIR). (**A**) shows a replication fork with a nick in a template strand; (**B**) The fork collapses upon running into the nick; (**C** to **E**) As in SDSA, the 5'-end is resected to give a 3'-overhang that invades homologous sequence and functions as a primer for extension by DNA polymerase. In BIR, both leading and lagging strands are synthesized [17] (not shown); (**F** and **G**) The extended molecule separates from its template and, failing to encounter another broken end, reinvades and is extended further. This cycle might be repeated several times. Eventually, a fully processive replication fork is formed (**H**) and replication is completed (**I**). Conventions in the diagram are as in Figure 1.

Heteroduplex can be formed during the repair of collapsed (broken) replication forks by break-induced replication (BIR) (Figure 3) [7]. In this case, one arm of the replication fork has broken off, so that there is a single double-strand end. This end is processed as in Figure 1 and coated with Rad51 protein. It then invades homologous duplex DNA and forms a low-processivity replication fork that separates (Figure 3F), as in SDSA. When the separated extended end does not encounter a complementary 3'-end from the other side of the break (because there is none) it reinvades, and eventually forms a fully processive replication fork that proceeds to the telomere or to the end of the replicon. As with SDSA, BIR predominantly uses the sister chromatid as a template, leading to no genetic consequence, but BIR can also occur with a homologous chromosome or with ectopic homology [8], giving the possibility of forming heteroduplex and subsequently causing conversion as described above. Re-establishment of a replication fork on the sister chromatid by BIR is shown in Figure 3. Because BIR can switch templates to a homologous chromosome [8], a substantial length of sequence, even as far as the telomere, can be transferred non-reciprocally. Thus BIR with non-sister DNA constitutes another mechanism of gene conversion. 

**Figure 4:**
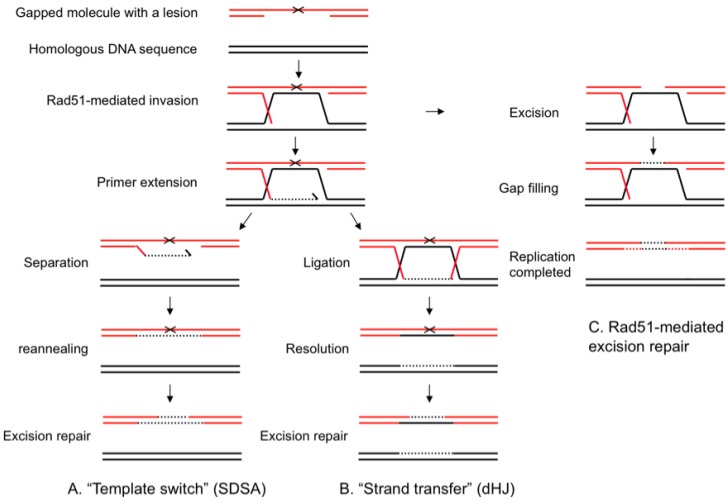
Recombinational mechanisms of gap-repair. A single-strand gap opposite a non-replicating lesion can be repaired by recombination using a homologous DNA molecule. The polymerase filling the gap can switch to the donor template (black), while the displaced donor strand anneals in the gap on the recipient molecule, so that a D-loop is formed in a reaction similar to Rad51-mediated DSB repair (Figure 1). (**A**), in template-switching gap repair, the newly synthesized strand is separated from its template, as in SDSA, and then re-anneals with the damaged strand. This allows removal of the lesion by excision repair; (**B**), the double Holliday junction formed by the switch is resolved by endonuclease or by helicase with topoisomerase as in dHJ recombination, again allowing excision repair; (**C**), Rad51-mediated excision repair uses a Rad51-mediated invasion to form a D-loop. The displaced strand of the D-loop anneals with the region containing the lesion, and provides a template for excision repair. In all three cases, sequence from the donor molecule (black) has been transferred to the damaged molecule (red) so that any allelic differences would have been transferred by conversion from the donor to the damaged molecule. The lesion is shown as an X. Other conventions are as in Figure 1.

Single-strand gaps occur after replication when a replication-blocking lesion in a template strand causes leading and lagging-strand synthesis to become uncoupled [9]. Thus the gap is opposite to a lesion and cannot be repaired by a normal gap-filling DNA polymerase. Similarly, a gap caused by excision repair might occur with a lesion in the remaining strand. In these situations, the gaps might be repaired by recombination. The mechanism has been interpreted as transfer of a complementary strand from a homologous sequence, or by a template switch during DNA synthesis that copies the relevant sequence from a homologous region and then transfers it back to the damaged molecule [10]. The same mechanisms have been found to be active in human cancer cell lines [11]. Both mechanisms are capable of generating heteroduplex DNA with consequent conversion. Figure 4 shows how both template switching and strand transfer can readily be understood in the context of the processes that we already understand for recombinational DSB repair. “Template switching” is analogous to SDSA and “strand transfer” is analogous to dHJ recombination. A further mechanism has been suggested by which a D-loop is formed and the displaced strand anneals with the lesion-carrying strand in the gap, allowing excision repair to remove the lesion so that replication can continue [12,13]. As shown in Figure 4C, this will convert any allelic difference near the lesion from the donor to the damaged molecule. 

### 2.3. Conversion during Meiosis

Gene conversion in meiosis is a well studied subject because of the availability of organisms in which the four products of a single meiosis are held together in a tetrad of cells (reviewed in [14,15,16]). This allows the non-reciprocity (unidirectional nature) of events to be seen directly. Conversion is strongly correlated with crossing-over, which is much more frequent than in mitotic cells, but half or more of the conversions are non-crossover events. Many conversion tracts originate outside genes and extend for a variable distance into genes from one or both ends. Co-conversion of adjacent alleles is also seen, supporting the concept that the conversion mechanism concerns a length of sequence. In many studies, the length of the meiotic conversion tract appears to be less than the length of a gene. Conversion of both strands of one DNA molecule is common, but there is also evidence of persistence of heteroduplex DNA, seen as post-meiotic segregation of alleles. This is often seen as being specific to certain alleles, perhaps because different mismatches in heteroduplex DNA are repaired differently by MMR, and only unrepaired mismatches produce post-meiotic segregation. Most conversion is seen in one or two of the four chromatids of a bivalent chromosome, and, where there is a crossover, it involves the converted chromatids. 

Meiotic conversion occurs by DSB repair by the same mechanisms as repair of DSBs in mitotic cells, although the specific proteins involved may differ [4]. Double-Holliday junction events predominantly generate crossovers, needed for proper chromosome segregation, and SDSA events generate non-crossovers [18] (Figure 1). The DSBs that initiate meiotic recombination are induced by a topoisomerase-like reaction mediated by Spo11 endonuclease (reviewed by [19]). These DSBs tend to occur between genes [20,21]. Genome-wide analysis of meiosis shows that, in yeast, 1% of the genome is being converted in any one meiotic cell [22]. Although sister chromatid exchange does occur in meiosis, recombination is mostly between homologous chromosomes, which are held together by synaptonemal complex, a protein structure that hold homologues in allelic register during the pachytene period of prophase of the first meiotic division (reviewed by [23]). Ectopic recombination events are less common than interactions between homologues, but they do occur at significant frequencies [24]. 

## 3. Mechanisms That Control Ectopic Conversion

### 3.1. Synaptonemal Complex

Synaptonemal complex is a highly ordered structure that is formed between homologous chromosome pairs during pachytene of the first meiotic prophase. The function of synaptonemal complex is not clear. It is unlikely that it functions to bring homologues together to facilitate recombination, or that it serves to ensure that ectopic recombinational reactions do not occur, because recombination intermediates form before synaptonemal complex (reviewed in [23]). Most likely is that synaptonemal complex functions to reject ectopic recombination events before they are finalized into the crossovers that provide chiasmata that regulate orderly separation of homologous chromosome pairs [23,25]. SDSA might be the mechanism by which excess recombination is aborted [26,27]. As described above, SDSA leaves the donor molecule intact, but transfers some donor information to the broken molecule. Thus a considerable amount of conversion between ectopic sequences can occur in very early prophase before the chromosomes are synapsed into correct homologous pairs. Indeed, recombination in yeast meiosis with homology on heterologous chromosomes is only one order of magnitude less frequent than allelic interactions [24]. 

### 3.2. Homology Requirements

Ectopic recombination is strongly limited by the length of sequence identity in non-allelic sequences because homologous recombination does not occur when the length of identity is short. The concept of minimal efficient processing segment (MEPS) was introduced to describe this phenomenon in *E. coli* [28]. Between 23 and 90 base-pairs of sequence identity were required, depending on which recombination pathway (set of recombination proteins) was assayed. Thus allelic differences interrupt a MEPS, and recombination between homeologous sequences (sequences with less than 97% identity) is initiated in those regions that do have sufficient length of identity [29]. A MEPS length of between 132 and 232 bp of identity was estimated for mouse [30,31], and a value of 337 to 456 was found for human [29] (reviewed by [32]).

A MEPS might reflect the ability of a recombinase such as Rad51 to initiate a recombination event and form a D-loop. However, it appears that a major constraint on homeologous recombination is enforced by the MMR system, which does not allow persistence of heteroduplex that contains mismatched base-pairs [31,33,34,35]. Even one mismatch has an effect, and numerous mismatches make recombination rate negligible [32]. This can be expected to have an impact on the length of duplicated sequence that allows recombination, because branch migration of a Holliday junction, which extends heteroduplex, will instigate MMR, and hence rejection of recombination, if it extends into heterologous sequence at the end of a duplicated segment. It might be useful to regard the MEPS as the length of identity required to form an event large enough to have stability, *i.e.*, to be independent of dissolution by MMR [32,34,36,37,38].

### 3.3. Chromatid Cohesion

A ring-like protein complex called cohesin holds sister chromatids together from S-phase until the chromatids separate at anaphase. This ensures the orderly segregation of sister chromatids [39,40,41]. Cohesins are then degraded by proteolysis to allow anaphase separation. Cohesin is recruited to sites of DNA damage [39,40], is necessary for DSB repair [42] and enforces physical proximity of the sisters (reviewed by [43]). Cohesin has been shown to restrict recombinational repair of damage from ionizing or ultra-violet radiation to interactions between sisters [44]. Consequently, recombination between homologous or ectopic sequences is rare in mitotic cells. 

Cohesins are also present during meiosis, where they hold sisters together until late prophase, when cohesion is aborted along chromosome arms, but not near centromeres where it is protected. This allows each pair of sister centromeres to behave as a unit held together through first division anaphase. Sister chromatid cohesion is thought to be relaxed locally during DSB-mediated recombination in meiosis, allowing the DSB to interact with the homologue instead of the sister [23]. 

## 4. Conclusions

Double-strand break-repair in mitotic cells is regulated by the action of cohesin to occur predominantly between sister molecules, reducing ectopic interactions. Ectopic recombination is also reduced by the requirement for an extensive length of identical sequence. Recombination between diverged sequences is prohibited by the mismatch repair system. 

However, the numerous DSBs made in early meiotic prophase open the cell to rampant recombination, and this recombination is constrained to favour interactions between homologues rather than sister molecules. In addition, many of the early recombinational interactions involve ectopic sequences. Much of this excess recombination is aborted, possibly by being channeled into the SDSA pathway, and the aborted events include the ectopic events: a reaction that is perhaps mediated by synaptonemal complex. The few recombination events that are not aborted mature into the crossovers needed to ensure orderly segregation. However, SDSA is a mechanism that frequently leads to conversion, so that the trace of these aborted events remains as conversion events, many of them ectopic. 

I suggest that a reasonable conclusion from these considerations is that ectopic gene conversion is a high probability outcome of the normal processes of meiosis. In contrast, ectopic conversion in mitotic cells appears to be an aberration caused by the failure of systems that guard against it. 
